# Structural dataset for the PPARγ V290M mutant

**DOI:** 10.1016/j.dib.2016.03.082

**Published:** 2016-04-04

**Authors:** Ana C. Puhl, Paul Webb, Igor Polikarpov

**Affiliations:** a Instituto de Física de São Carlos, Universidade de São Paulo, Av. Trabalhador Saocarlense 400, São Carlos, SP 13560-970, Brazil; b Genomic Medicine Program, Houston Methodist Research Institute, Houston, TX, United States

**Keywords:** Peroxisome proliferator activated receptor γ, Nuclear receptors, X-ray structureç ligand resistance syndrome

## Abstract

Loss-of-function mutation V290M in the ligand-binding domain of the peroxisome proliferator activated receptor γ (PPARγ) is associated with a ligand resistance syndrome (PLRS), characterized by partial lipodystrophy and severe insulin resistance. In this data article we discuss an X-ray diffraction dataset that yielded the structure of PPARγ LBD V290M mutant refined at 2.3 Å resolution, that allowed building of 3D model of the receptor mutant with high confidence and revealed continuous well-defined electron density for the partial agonist diclofenac bound to hydrophobic pocket of the PPARγ. These structural data provide significant insights into molecular basis of PLRS caused by V290M mutation and are correlated with the receptor disability of rosiglitazone binding and increased affinity for corepressors. Furthermore, our structural evidence helps to explain clinical observations which point out to a failure to restore receptor function by the treatment with a full agonist of PPARγ, rosiglitazone.

**Specifications Table**

**Table:** 

Subject area	*Biology*
More specific subject area	*Protein Crystallography, Biochemistry, Nuclear Receptors*
Type of data	*Table, figures, text file, binary files*
How data was acquired	*Advanced Light Source (ALS), beamline 5.0.1 - Lawrence Berkeley National Laboratory (Berkeley, CA, USA)*
Data format	*PDB format text file. Binary structure factors in mtz format. Analyzed by iMosflm, Phenix and Coot and Pymol*
Experimental factors	PPARγ V290M LBD at 15 mg/mL mixed with 1 mM of sodium diclofenac on ice and allowed to stand at 277 K overnight prior crystallization. Crystals were soaked in a cryoprotectant containing the same reservoir solution added with 15% (v/v) ethylene glycol and rapidly cooled in a gaseous nitrogen stream at 100 K for data collection.
Experimental features	PPAR*γ* LBD V290M mutant was *crystallized by sitting drop vapor diffusion, cooled to 100* *K and subjected to synchrotron radiation from which X-ray diffraction images were collected. The images were computationally analyzed to index reflections and extract associated intensities. Molecular replacement technique was used to obtain phase information. Final structure model was obtained* after several rounds of a combination of automated and manual refinement.
Data source location	*Berkeley, CA, USA*
Data accessibility	*Data is within this article. Protein coordinate data is publically available is the RSCB Protein Database (http://www.rscb.org/) using the PDB: 4OJ4*

**Value of the data**

• PPARγ LBD V290M mutant [1] is one of the mutants associated with PPARγ Ligand Resistance Syndrome [2], [3], [4]. People harboring this mutation do not respond to a treatment with full agonists such as rosiglitazone. Structure provides explanation for the impairment of PPARγ function and decreased affinity to full agonists.
• Structure of PPARγ LBD V290M mutant in complex with diclofenac shows that partial agonists can bind to PPARγ receptor mutants.
• Structure of PPARγ LBD V290M mutant may assist in designing of new compounds that have a capacity to bind to the mutant receptor and might offer new possibilities of medical treatment for patients harboring this mutation.

## 1 Data

The present article provides details on crystallization, data collection and structure determination of PPARγ V290M mutant (PDB: 4OJ4). We also describe the mutant receptor structure and how this mutation could impair PPARγ function.

## 2 Experimental design, materials and methods

### 2.1 Site directed mutagenesis, protein expression and purification

Mutagenesis was performed using the PET28a vector containing PPARγ LBD nucleotide sequence. The primer CATTATTCTCAGTGGAGAC**T**GCCCAGGTTTGCTGAATGTG was used to substitute amino acid residue valine 290 by methionine (V290M) using standard site-directed mutagenesis with the *QuickChange site-directed mutagenesis kit* (Stratagene). The mutation V290M was verified by sequencing. The plasmid pET28a(+) (Novagen) encoding a human PPARγ V290M LBD fused in frame to the C-terminus of a polyhistidine (His) tag was used to transform competent *Escherichia coli* strain BL21 (DE3). The expression and purification of PPARγ V290M LBD was performed as described [7].

### 2.2 Crystallization, data collection and structure determination PPARγ V290M mutant

Several strategies were used to crystallize PPARγ V290M LBD by varying protein concentration (5–18 mg/mL), temperature (277 and 291 K), crystallization techniques (hanging drop, sitting drop, microbatch and seeding) and by addition of ligands (sodium diclofenac, luteolin, nonanoic acid, rosiglitazone and troglitazone), detergents and additives (Hampton Research). The successful conditions for crystallization were obtained using PPARγ V290M LBD at 15 mg/mL mixed with 1 mM of sodium diclofenac (Sigma Aldrich) on ice and allowed to stand at 277 K overnight. The crystallization screens were performed using a Mosquito (TTP LABtech) by sitting drop of 0.5 µl of protein complex solution mixed with 0.5 µl precipitant solution and equilibrated against a 100 mL reservoir solution from several commercial kits. Crystals were obtained with PEG 4000 30%, CaCl_2_ 200 mM and HEPES 100 mM pH 7.5 (Kit PEG II, Qiagen). Before data collection, the crystals were soaked in a cryoprotectant containing the same reservoir solution added with 15% (v/v) ethylene glycol and rapidly cooled in a gaseous nitrogen stream at 100 K. X-ray diffraction data were collected in the protein crystallography beamline 5.0.1. of the Advanced Light Source (ALS) at Lawrence Berkeley National Laboratory (Berkeley, CA, USA). Diffraction data were processed using MOSLFM [11] and scaled with SCALA from the CCP4 program suite [12]. The structure was determined by molecular replacement using the program PHASER from CCP4 Packages and the PPARγ LBD structure (PDB: 3SZ1) as a model removing H12, in which mutation V290M is located. The programs PHENIX and COOT were used to alternately run cycles of refinement and model building [13], [14].

**Fig. 1:**
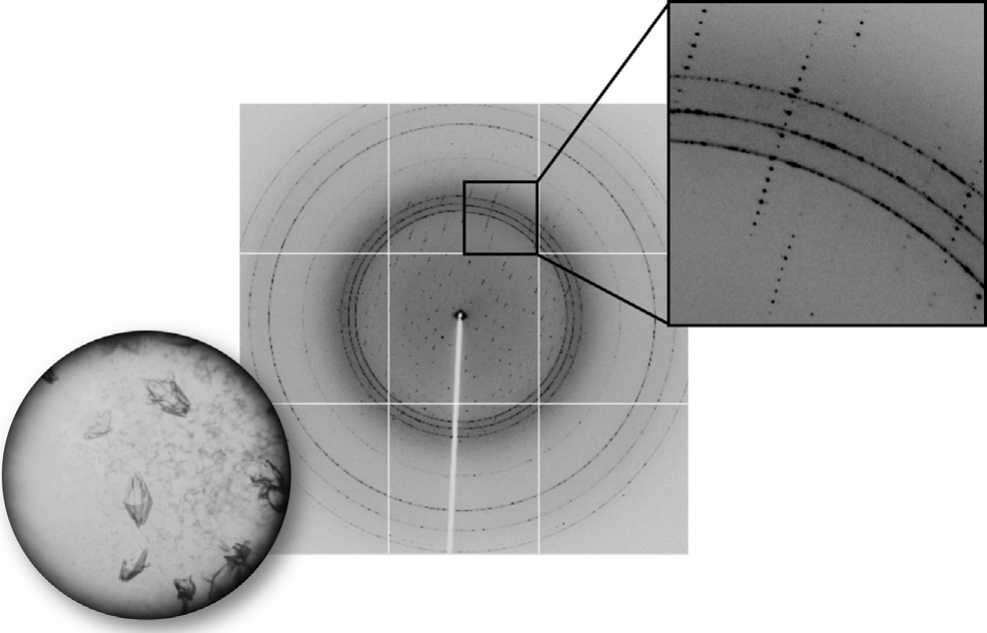
Crystals and X-ray diffraction image from the PPARγ V290M mutant dataset collected at (ALS), beamline 5.0.1.

We crystallize the mutant PPARγ LBD with an anti-inflammatory compound that behaves as a PPARγ partial agonist [5]. The X-ray diffraction dataset for PPARγ V290M mutant consist of 360 diffraction images that were collected at 0.5° rotation of the crystal giving 180° of diffraction data. The diffractions spots are clean sharp and show a single lattice. There is also an evidence of crystalline water (ice) formation (Fig. 1).

The PPARγ V290M mutant crystals belong to the space group P4_3_2_1_2 with the unit cell dimensions *a*=*b*=60.04 Å, *c*=161.19 Å and diffracted to 2.3 Å resolution. The 12,961 unique reflections were measured with an overall redundancy of 7.2. All data processing statistics indicate a high quality of the dataset (Table 1). After molecular replacement and several rounds of intercalated automated refinement and manual model building, the PPARγ V290M crystal structure consisting of 2105 non-hydrogen atoms was built. The structure was refined to R-work=21.94 %, R-free=28.37% and has an average B-factor of 47.48 Å^2^. Table 1 summarizes the final data processing and refinement statistics.

### 2.3 Crystal structure of PPARγ LBD V290M mutant

#### 2.3.1 Electron density map and diclofenac binding site

**Fig. 2:**
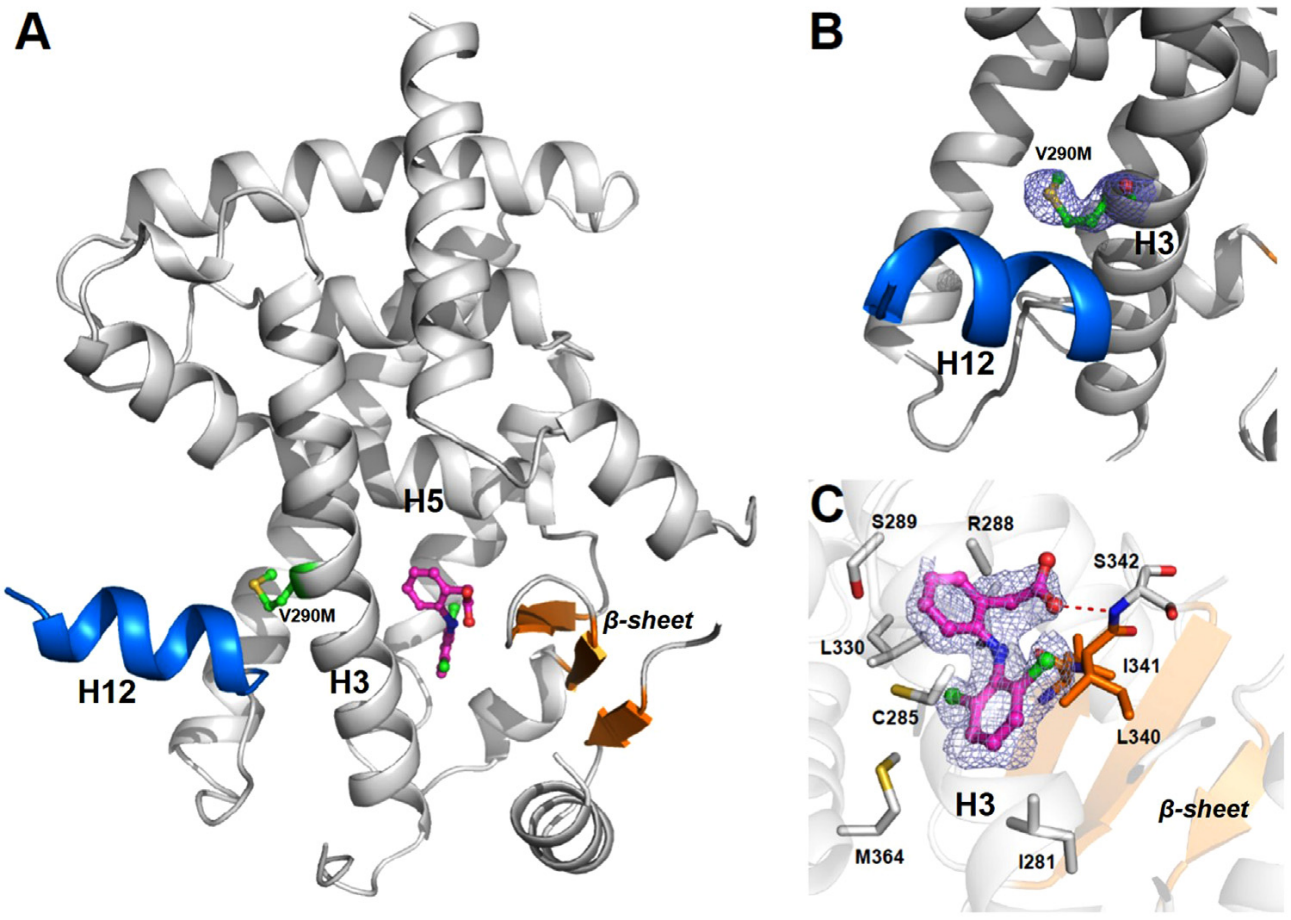
(A) Crystal structure of PPARγ LBD V290M mutant. H12 is colored in blue and β-sheet in orange (B) Omit map 2Fo–Fc is shown in a contour of 1.0 *σ* for the region around methionine M290 (green, *stick ball*). H12 is colored in blue. (C) Omit map 2Fo–Fc is shown in a contour of 1.0 *σ* for the diclofenac (pink, *stick ball*). Residues that interact with diclofenac are shown as sticks and β-sheet in orange.

A single PPARγ LBD forms the asymmetric unit of this 2.3 Å crystal structure, which is different from most other PPARγ structures that frequently display two juxtaposed LBDs (A and B subunits) in the asymmetric unit, even though in the present PPARγ V290M structure the dimeric arrangement is realized by the interaction with a symmetric related monomer. The PPARγ V290M LBD adopts standard NR fold and consists of 12 α-helices that form the classic three-layered antiparallel β-helical sandwich fold with a small four-stranded β-sheets [6], [7] (Fig. 2). The electron density was easily interpretable and 2fo–fc map where diclofenac and the residue 290 were omitted showed strong density corresponding to the mutant residue methionine and the presence of diclofenac at the binding pocket. Diclofenac adopts a binding mode that resembles that of other partial agonists, lying in a north-south orientation close to helix 3 and the β-sheets, in a similar position described for the wild type receptor [5]. The only major differences between previous structures of the wild type PPARγ with agonists and partial agonists and the mutant PPARγ LBD were related to helix H12 position and we therefore will focus upon this particular aspect of the structure in our further analysis.

### 2.4 Crystal packing and structural changes

**Fig. 3:**
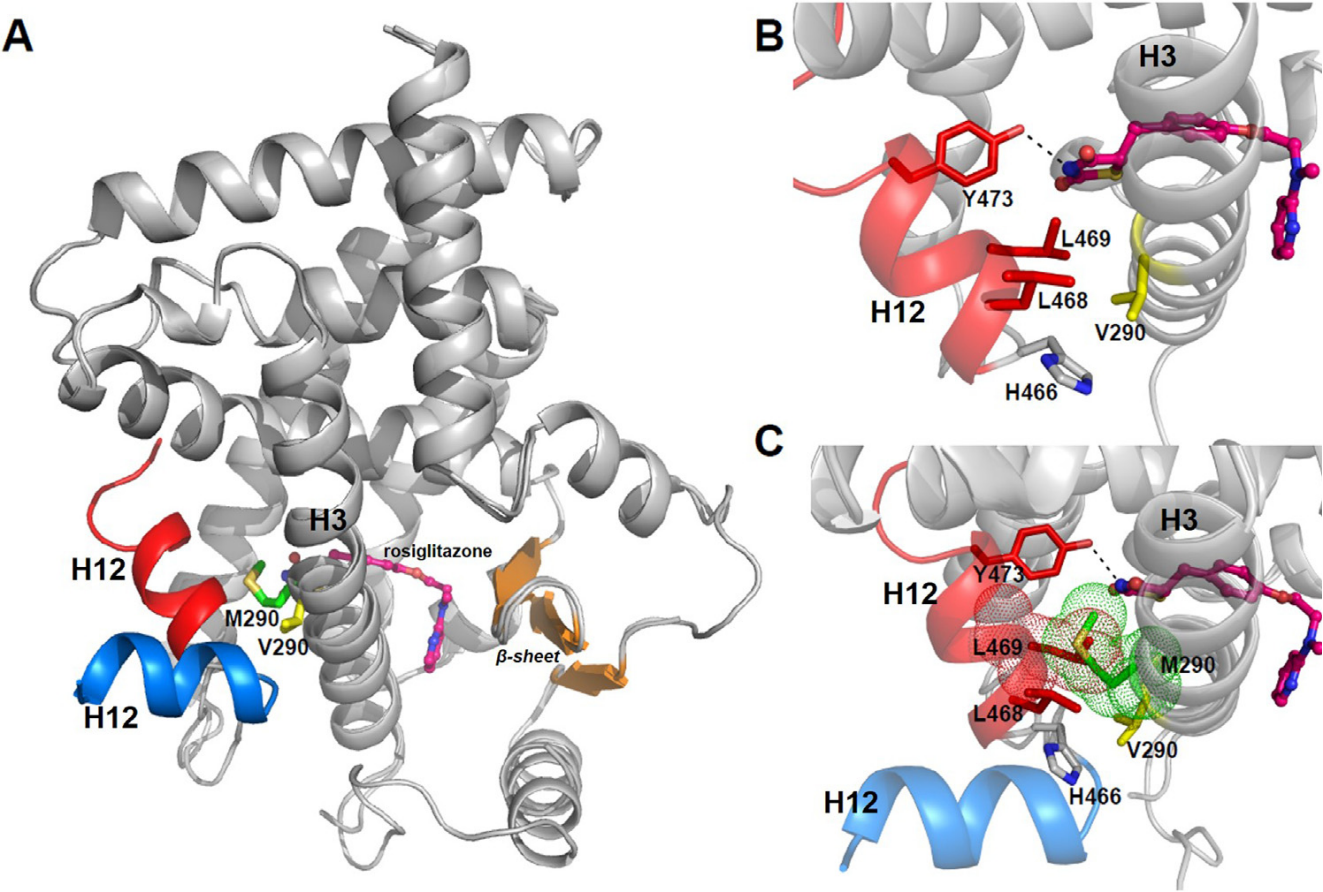
Comparisons of the crystal structures PPARγ LBD V290M (PDB: 4OJ4) and 2PRG structures (Diclofenac from PDB: 4OJ4 was omitted for clarity) (A). Valine 290 is an important residue for correct positioning of H12 and make hydrophobic interactions with residues L468, L469 and H466. Y473 from H12 makes a hydrogen bond with rosiglitazone, stabilizing this helix in an active conformation (B). Methionine M290 is a bulky residue than valine and cause clashes with L469, dislocating H12 and preventing its closure. H12 from PPARγ LBD with rosiglitazone (pink, stick ball) (PDB: 2PRG) is shown in red and H12 from mutant V290M structure is shown in blue. (C) H12 from PDB: 2PRG is shown in red and H12 from V290M structure (PDB:4OJ4) is show in blue. Rosiglitazone from PDB: 2PRG is shown in pink (stick ball). Residues L469 and M290 are shown as dots and represent Van der Waals interactions.

Loss-of-function mutation V290 M in the ligand-binding domain of PPARγ is associated with a syndrome characterized by partial lipodystrophy and severe insulin resistance [1]. In the structure we describe here, H12 protrudes from the V290 M mutant receptor surface at almost 90^o^ angle (Fig. 2 inset). This is different from the standard active conformation typified by a PPARγ structure with rosiglitazone and the coactivator SRC1 peptide bound to AF-2, where H12 packs against the H3 and H5 to complete the coactivator binding surface (PDB: 2PRG) [6] (Fig. 3A). It is likely that steric clashes between the methionine substituent and H12 are responsible for altered H12 position and orientation. V290 is located on helix H3 and plays an important role in H12 positioning, making hydrophobic interactions with H12 H466, L468 and L469 (Fig. 3B). The bulky side-chain of methionine (Fig. 3C) would clash with the L469 side-chain, preventing H12 closure. Additionally, L469 contributes to the accommodation of the polar head of TZDs by hydrophobic interactions and Y473 makes a hydrogen bond with the nitrogen of TZDs (Fig. 3C). Thus, it is also likely that blockade of H12 closure will disrupt interactions with rosiglitazone and other TZDs and will impair TZD-dependent stabilization of the domain.

It is likely that aberrant H12 position in the PPARγV290M mutant would affect recruitment of coactivators. Correct H12 position is important for binding of short LxxLL motifs present in coactivators such as SRC1 and CBP [8]. Agonists stabilize H12 to form a hydrophobic cleft that docks Leu residues of LxxLL motif and also positions H12 E471 to complete a charge clamp that helps to anchor the coactivator peptide via interactions with the helix dipole [6]. Superimposition of coactivator peptide SRC1 from structure PDB: 2PRG (data not shown), reveals clashes between the coactivator lysine and H12 from the mutant receptor suggesting that coactivator binding would be precluded in this conformation. Besides this, the destabilization of H12 caused by mutation V290M could also impair binding and activity of other agonists that interact with H12, such as NSAIDS [5]. However, it was shown that some tyrosine-based (TA) receptor agonists can rescue mutant receptor function [9]. TAs (*e.g.* farglitazar) corrected defects in ligand binding and coactivator recruitment by natural dominant negative PPAR mutants (V290M and P467L), restoring transcriptional function comparable with wild-type receptor. More complete ligand-dependent corepressor release and reversal of dominant-negative inhibition was achieved with TA than TZD agonists, that could be explained because farglitazar makes more extensive contacts than rosiglitazone within the ligand-binding pocket, to stabilize helix 12, facilitating corepressor release and transcriptional activation [9].

**Fig. 4:**
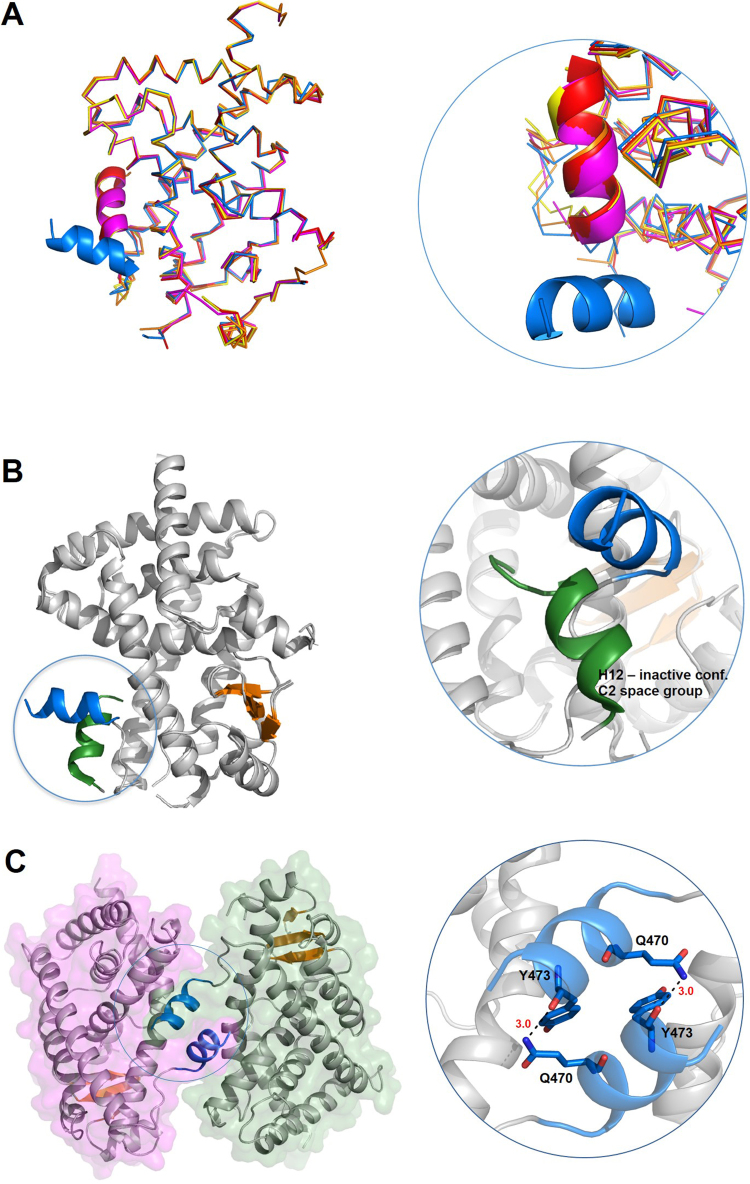
(A) Superimposition of crystal structure of the mutant V290M (PDB: 4OJ4, H12, blue) with structures 3BCU (magenta), 3PRG (orange), 3R5N (red), and 4XLD (yellow) showing that the H12 in the V290M adopts a different conformation in comparison of active conformation found in the structures crystallized in the same space group P43212. (B) Superimposition of crystal structure of the mutant V290M (PDB: 4OJ4, H12, blue) with that of the chain B in the complex PPARγ/luteolin (PDB3SZ1), showing H12 (dark green) indifferent inactive conformation. Both structures show inactive conformation of H12 and both are due to crystal packing (C) Analysis of the crystal packing of the mutant V290M showing that the conformation adopted by H12 occurs because hydrogen bonds between Q470 and Y473 with the H12 of symmetric related monomer.

PPARγ usually crystallizes in the C2 space group with two LBDs in the asymmetric unit; an A-subunit that adopts active conformation irrespective of the presence and type of ligand (agonist, partial agonist) and a B-subunit that adopts an inactive conformation with H12 displaced from the body of the domain. Novel H12 position observed in the present PPARγV290M structure also differed from the wild type PPARγ B subunit (Fig. 4B). Here, however, differences in H12 position could partly reflect influences of crystal packing. We compared H12 of our structure with other four structures that belong to the same space group P4_3_2_1_2 (PDB: 3BCU, PDB: 3PRG, PDB: 3R5N, and PDB: 4XLD (Fig. 4A). For the PPARγV290M mutant, H12 docks against its counterpart in the symmetric equivalent neighboring monomer (Fig. 4C), whereas H12 from the wild type PPARγ B subunit docks into the AF-2 site of the A subunit of the neighboring dimer. Unusual H12 position of PPARγ was also detected in the structure of the mutant F360L (PDB: 4L98), associated with familial partial lipodystrophy, showing a similar interaction between H12 facing monomers and was attributed to crystal packing [10] .

## Figures and Tables

**Table 1:** X-ray crystallographic data collection and refinement statistics.

**Data collection**		**Refinement**	
**Source**	APS 5.0.1	**Resolution**	48.19–2.3 (2.36–2.3)
**Wavelength**	0.999 A°	**Reflections used**	12210
**Space group**	P4_3_2_1_2	**Reflections for R-free**	643
**Unit cell parameters**		**Non-hydrogen atoms**	2105
a**(A°) = b (A°)**	60.04	**Protein**	2026
c**(A°)**	161.19	**Diclofenac/ Calcium**	21
**Molecules in the ASU**	1	**Water**	58
**Resolution (A°)**	56.26-2.3 (2.42-2.3)	**R_*factor*_**^c^**(%)**	21.94
**Unique reflections**	12,961 (1,950)	**R_*free*_**^d^**(%)**	28.37
**Redundancy**	7.2 (7.6)	**rmsd bond lenghts (A°)**	0.013
**Completeness (%)**	93.2 (100)	**rmsd of bond angles (°)**	1.638
**R_*sym*_**^b^	0.152 (0.625)	**Average B-factor (A°^2^)**	47.48
**<*I*>/<*σ(I)*>**	7.4 (2.80)	**Ramachandran *outliers***	1
**Wilson B-factor (A°^2^)**	36.3	**Molprobity score**	1.79 (96%)
		**PDB code**	PDB: 4OJ4

a Values in parameters refer to the last resolution shell.

b *R*_*sym*_: *∑*_*hkl*_*,*_*j*_ (|I_hkl_–<I_hkl_>|)/*∑*_*hkl*_*,*_*j*_ I_hkl_, where <I_hkl_> is the average intensity for a set of *j* symmetry-related reflections, and %.

c *R*_*factor*_*: ∑*_*hkl*_ (*||F*_*o*_*|–|F*_*c*_*||*)*/∑*_*hkl*_*|F*_*o*_*|,* where *F*_*o*_ is the observed structure factor amplitude, and *F*_*c*_ is the calculated structure factor amplitude.

d *R*_*free*_*: ∑*_*hkl*_*,T(||F*_*o*_*|–|F*_*c*_*||)/∑*_*hkl*_*|F*_*o*_|, where a test set, *T* (5% of data), is omitted from the refinement.
